# Assessment of Mongolian dietary intake for planetary and human health

**DOI:** 10.1371/journal.pgph.0001229

**Published:** 2023-03-01

**Authors:** Dashzeveg Delgermaa, Miwa Yamaguchi, Marika Nomura, Nobuo Nishi

**Affiliations:** 1 Department of Public Health Policy Implementation Coordination, National Center for Public Health, Ministry of Health, Ulaanbaatar, Mongolia; 2 International Center for Nutrition and Information, National Institute of Health and Nutrition, National Institutes of Biomedical Innovation, Health and Nutrition, Tokyo, Japan; 3 Human Development Department, Japan International Cooperation Agency (JICA), Tokyo, Japan; Nepal Health Research Council, NEPAL

## Abstract

**Background:**

Healthy diets that consider environmental pressures are required to meet sustainable development goals in Mongolia. This study aimed to clarify the extent of planetary and human health on Mongolian dietary intake.

**Methods:**

The intake of eight food groups (g/day) was investigated using the national database of the Household socio-economic survey (HSES) 2019 in Mongolia. The boundary intake of the Planetary health diet (PHD) proposed by the EAT-Lancet Commission was considered 100% adequate. The adequacy (%) of food groups in the HSES were calculated in two areas (urban and rural), during the two seasons (cold and warm), and the total by each boundary of the PHD. The differences between the recommended dietary intake (RDI) in Mongolia and the PHD were also investigated in the same manner.

**Results:**

The adequacy of red meat (i.e., beef, mutton, and horsemeat) in whole areas of Mongolia indicated more than 17 times higher intake (1,738%) than the PHD. The adequacy of vegetables (20%) and fruits (8%) in Mongolia indicated an intake shortage compared to the PHD. These discrepancies in dietary adequacy were greater in rural areas and during the cold seasons than in urban areas and during the warm seasons, respectively. The animal-based protein sources, especially red meat (1,091%), in the RDI of Mongolia were higher than those in the PHD.

**Conclusion:**

This study found a highly excessive intake of red meat and a low intake of vegetables and fruits compared with the PHD among Mongolian people, especially in rural areas and during the cold seasons. The limited diversity of food in severe geographic conditions, poor accessibility of food retailers, and insufficient nutrition education may have led to these results. Therefore, improvements in the food environment and nutritional education are required.

## Introduction

Food production negatively influences global environmental change, which threatens food security at the same time [1]. Mongolia has experienced noticeable climate change, with an increase in the average temperature of ≥ 2°C and a decline in rainfall between 1940 to 2015 [2]. Extreme weather, such as drought in summer, results in the loss of livestock and threatens food security and the population’s health [2]. Food production generates greenhouse-gas (GHG) emissions, nitrogen and phosphorus pollution, biodiversity loss, and water and land use [3]. The global GHG emissions from food production include 57% from animal-based food (including livestock feed), 29% from plant-based foods, and 14% from other uses [4].

In addition to securing natural resources, dietary habits are also important when considering population health. In Mongolia, the prevalence of obesity (20.9% in men and 26.5% in women) among adults aged 18 years and older is higher than in the Asian region (17% in men and 20% in women), in addition to the double burden of malnutrition among children and women [5]. Overconsumption of animal-based foods may threaten population health. A review indicated that total protein and animal protein were associated with the risk of cardiovascular diseases and diabetes [6]. Prospective cohort studies and meta-analyses reported that total protein intake was positively associated with all-cause mortality, and higher animal protein intake was associated with mortality from cardiovascular disease [7].

The EAT-Lancet Commission proposed the Planetary health diet (PHD) [3], a framework of planetary boundaries that indicates the intake ranges of food groups to ensure human health and environmental sustainability. The framework recommends the predominant consumption of plant-based foods (vegetables, greens, fruits, and whole grains) and small amounts of animal-based foods (meat, fish, and eggs). This framework has been used in several studies. Studies in Brazil developed the PHD index, confirmed its validity and reliability [8], and found that high adherence to the index was associated with a lower prevalence of obesity [9]. A study in India reported that people consumed cereals, fruits, and vegetables but not enough protein compared with those in the PHD [10]. A study in Denmark proposed the development of a Danish diet adapted to a healthy plant-based diet aligned with the PHD [11]. The benefits of the PHD have been reported not only for health but also for socio-economic reasons. For example, an Australian study reported that a PHD basket was less expensive and more affordable than a typical Australian diet basket [12].

Although some countries have assessed their diets based on the PHD, there is no evidence of the PHD in Mongolia. Traditional Mongolian food is based on the products of nomadic animal herders who raise Mongolian steppe meat and milk [13]. Nomadic culture continues to be practiced in rural areas. Dietary habits in urban areas changed with socio-economic growth, and dietary patterns were positively associated with body mass index [13]. Food consumption is seasonal, particularly in rural areas. Dairy products and meat are highly consumed during winter [14]. In this context, assessing Mongolian diets in urban and rural areas and during cold and warm seasons is needed from the perspective of both planetary and human health.

Therefore, this study aimed to clarify how Mongolian dietary intake was aligned with the PHD using a national survey and compare it between areas (i.e., urban and rural) and seasons (i.e., cold and warm). In addition, to understand the sustainability of national dietary recommendations, this study investigated the differences between national dietary targets and the PHD.

## Materials and methods

### Study setting

Mongolian dietary intake was investigated using open-source data from the Household socio-economic survey (HSES) performed in 2019 [15]. The health boundary of the PHD was set as the benchmark for dietary intake. The recommended dietary intake (RDI) in Mongolia was used to assess the sustainability of the national dietary recommendations [17].

### Household socio-economic survey

#### Target population

The HSES is a nationally representative survey that estimates and monitors a country’s level of poverty and people’s living standards. The HSES 2019 was conducted following the procedure of the HSES 2016 [15, 16].This survey used the sampling frame developed by the National Statistics Office, based on population figures obtained from administrative records.

#### Data collection

The 936 households were randomly surveyed each month from January 1st 2019 to January 1st 2020, for the HSES 2019, and a total of 11,232 households were selected. Of the total households, 11,197 participated in the survey (99.7% participation rate).

Urban and rural areas were classified according to the following steps. First, geographic domains were classified into four residential zones: Ulaanbaatar as an urban area, and as rural areas, 20 aimag (province) centers, 306 soum centers (i.e., a secondary subdivision outside Ulaanbaatar), and 891 Bags. Second, a primary sampling unit was selected in each zone using the probability proportional estimated size. Finally, 3,600 households in urban areas and 7,597 households in rural areas were randomly chosen from primary sampling units.

The HSES investigated dietary intake during the cold (October to March) and warm (April to September) seasons. However, there was no information on whether dietary intake during the two seasons was investigated in all 11,197 households.

#### Questionnaires

The core questionnaire of household socio-economic data and household food consumption was made according to the previous surveys [15, 16]. In the household socio-economic data, to indicate the socio-demographics, this study employed age (18 years and older), sex (men), number of household members (four [median] or more), type of dwelling (Ger), raised or owned livestock (herding, poultry, or any animal) (yes), owned agricultural land (yes), and household enterprise (yes). In the household food consumption, the field offices transmitted the data and provide additional clarification to a survey team in the National Statistics Office through field supervisors. The survey team in the National Statistics Office performed logical and consistent checks for all data. A representative household reported a dietary record and some households were asked to revise their answers whenever the field office found an error. A 30-day dietary record compiled by a researcher every 10 days, three times during a single month, was recorded to capture the household’s food consumption in urban areas. A 7-day dietary record was administered to all provinces in rural areas using the following question: “how much food item have you consumed in total during the past 7 days?” In both urban and rural areas, the household representative answered the question, “did you or any member of your household spend the following items during the past month/ or the past 7 days?” If the answer was “no,” the major place of “restaurants, cafes” or “canteens in schools, works canteens” was selected.

### EAT-Lancet reference diet

This study used the PHD as a reference diet to assess the dietary intake of Mongolian [3]. The PHD indicated the scientific targets and ranges of intake of 11 food groups (e.g., 300 g/day ranged from 200 to 600 g/day in all vegetables), added fats (unsaturated and saturated oils), and added sugars (all sugars) per 2,500 kcal/day of total energy. In addition, six food groups (beef, lamb, and pork; chicken and other poultry; eggs; fish; legumes; and nuts) were included as protein sources.

### Recommended dietary intake in Mongolia

The RDI in Mongolia indicates the daily target consumption of meat, meat products, flour, bakery products and various types of rice based on the average daily consumption in Mongolians [17]. The targets of total daily energy intake and energy from fat and nutrients (i.e., unsaturated and saturated fats) were referenced according to the human energy requirement in 2001/2002 proposed by the Ministry of Health in Mongolia [18].

### Food groups

This study classified food items of the HSES 2019 based on the food-based dietary guidelines “Ger” into the PHD food groups (Table 1) [19]. The national food guide is designed to shape a Mongolian wooden tent “Ger.” The food guide is divided into three food group layers: cereals and cereal products are placed at the bottom of the tent; vegetables, meat, fish, and eggs are placed at the second level; and fruits and dairy products are placed at the final level [19]. Although the EAT-Lancet Commission named the food group “beef, lamb, and pork” red meat [20], beef, mutton, horse meat, and camel meat are among the major red meats in Mongolia [15]. Therefore, this study changed the name to “red meat” to be understandable. Confectionery products and sugar were included in the “added sugar” in the PHD food group since the HSES indicated the intake of the two foods as one food group. We did not classify whole grains because the information was not available in the HSES.

**Table 1:** Classification of food items in the household socio-economic survey based on the planetary health diet.

Planetary health diets	Household socio-economic survey
Food group	Food item
Whole grains	Not available
Tubers or starchy vegetables	Potatoes, chips, and potato products
All vegetables	Green, yellow, and other vegetables
All fruits	Fresh fruit (e.g., wild fruit, apple, orange, kiwi, banana, pineapple, watermelon, seabucktorn, blueberry)
Dairy foods	Milk, cheese, curd, cream, Mongolian cheese, yogurt, mare milk, other dairy products, and other milks
Protein sources	
Red meat	Beef, mutton, horsemeat, goat meat, camel meat, pork, ham and sausages, other meats
Chicken and other poultry	Chicken and duck
Egg	Egg, other poultry eggs, and dried eggs
Fish	Fresh fish, salmon, white fish, canned fish, and smoked fish
Legumes	Corn, tofu, bean, peas, and preserved pea
Nuts	Tree nuts, ground nuts, walnuts, and any seeds
Added fats	
Unsaturated oils	Margarine and vegetable oils
Saturated oils	Butter, animal fats, lard, and suet
Added sugars	Sugar and confectionery products

### Data analysis

#### Socio-demographics

The proportion of people aged 18 years and older and men was calculated by individuals. The proportion of four or more household members, Ger of dwelling, raised or owned livestock, owned agricultural land, and household enterprise was calculated per household. The result was shown as a whole area because no data were available on the living areas (i.e., urban and rural) in which the houesholds lived.

#### Adequacy of dietary intake against the PHD

The health boundary and range (g/day) of each food group against 2,500 (kcal/day) proposed by the PHD were converted by the total energy intake (3,085 kcal/day) among Mongolian people aged 18 years and older. The adequacy (%) of each food group was calculated by dividing dietary intake (g/day) by the converted health boundary (g/day). Similarly, we calculated the health boundary, range, and adequacy in two areas (urban and rural), during the two seasons (warm and cold), and the RDI in Mongolia using each dietary (g/day) and energy intake (2,863 kcal/day in urban areas, 3,529 kcal/day in rural areas, 3,057 kcal/day during the cold season, 3,111 kcal/day during the warm season, and 2,400 kcal/day in the RDI in Mongolia). The food groups of chicken and other poultry, egg, fish, legumes, and nuts were not used in these two areas because this information was not presented in the database. For the same reason, this study did not use nuts in either season or RDI in Mongolia.

#### Ethics

This study used tabulated and published information on the HSES. The National Statistics Office obtained informed consent from the household representatives.

## Results

### Characteristics of the population

The 64% of individuals in the households were over 18 years (48% men) (Table 2). Over half of households lived with four or more members, and 41% of them lived in Ger. The 34% and 5.5% of households possessed the livestock and agricultural land, respectively. A household enterprise was present in 12% of households.

**Table 2:** Household characteristics in the household socio-economic survey.

Characteristics ^a^	N (%)
**Individual** ^**b**^	
Age, ≥ 18 years old	26182 (64)
Sex, men	19909 (48)
**Household**	
The number of household members, ≥ 4 ^c^	6015 (54)
Type of dwelling, Ger	4636 (41)
Raised or owned livestock (i.e., herding, poultry, or any animal), yes	3810 (34)
Owned agricultural land, yes	620 (5.5)
Household enterprise, yes	1318 (12)

^a^ The characteristics were referenced according to the Household socio-economic survey 2019 among 11,197 households.

^b^ Proportion of age and sex were calculated among 41,117 individuals among 11,197 households.

^c^ The median (four) of the number of household members was used.

### Dietary intake in whole and two areas in comparison to the PHD

Table 3 compares Mongolian dietary intake with the PHD in whole areas, urban areas, and rural areas. In whole areas, one of the major differences between the two diets was red meat which was more than 17 times higher intake (300 g/day, 1,738% adequacy) in Mongolia than that recommended by the PHD (17 g/day ranged from 0 to 35 g/day). On the other hand, all vegetables and fruits in Mongolia were lower (73 g/day, 20% adequacy in all vegetables and 20 g/day, 8.1% adequacy in all fruits) than these in the PHD (370 g/day ranged from 247 to 740 g/day in all vegetables and 247 g/day ranged from 123 to 370 g/day in all fruits). Other food groups indicated 100% and more adequacy (105–146% adequacy) but almost within the range of each PHD, except for unsaturated oils (13% adequacy). High intake of red meat and low intake of all vegetables and fruits were more evident in rural areas than urban areas.

**Table 3:** Mongolian dietary intake compared with the health boundary of the planetary health diet and the adequacy in total and two areas.

	Whole areas			Urban areas			Rural areas		
Food groups	Dietary intake (g/day)^a^	PHD boundary (range) (g/day)^b^	Adequacy (%)^c^	Dietary intake (g/day)^a^	PHD boundary (range) (g/day)^b^	Adequacy (%)^c^	Dietary intake (g/day)^a^	PHD boundary (range) (g/day)^b^	Adequacy (%)^c^
Whole grains	N.A.	286	N.A.	N.A.	266	N.A.	N.A.	328	N.A.
Tubers or starchy vegetables	90	62 (0, 123)	146	97	57 (0, 115)	169	83	71 (0, 141)	118
All vegetables	73	370 (247, 740)	20	87	344 (229, 687)	25	57	424 (282, 847)	14
All fruits	20	247 (123, 370)	8.1	23	229 (115, 344)	10	17	282 (141, 424)	6.0
Dairy foods	366	309 (0, 617)	119	273	286 (0, 573)	95	480	353 (0, 706)	136
Protein sources									
Red meat	300	17 (0, 35)	1738	247	16 (0, 32)	1540	367	20 (0, 40)	1963
Chicken and other poultry	N.A.	36 (0, 72)	N.A.	N.A.	33 (0, 66)	N.A.	N.A.	41 (0, 82)	N.A.
Egg	N.A.	16 (0, 31)	N.A.	N.A.	15 (0, 29)	N.A.	N.A.	18 (0, 35)	N.A.
Fish	N.A.	35 (0, 123)	N.A.	N.A.	32 (0, 115)	N.A.	N.A.	40 (0, 141)	N.A.
Legumes	N.A.	93 (0, 123)	N.A.	N.A.	86 (0, 115)	N.A.	N.A.	106 (0, 141)	N.A.
Nuts	N.A.	62 (0, 93)	N.A.	N.A.	57 (0, 86)	N.A.	N.A.	71 (0, 106)	N.A.
Added fats									
Unsaturated oils	6.6	49 (25, 99)	13	10	46 (23, 92)	22	6.7	57 (28, 113)	12
Saturated oils	16	15 (0, 15)	110	13	14 (0, 14)	96	20	17 (0, 17)	121
Added sugars	40	38 (0, 38)	105	43	36 (0, 36)	121	40	44 (0, 44)	92

N.A.: not available, PHD: Planetary Health Diet

^a^ The average dietary intake (g/day) was referenced according to the Household socio-economic survey (HSES) 2019 among 11,197 households (3,600 in urban areas and 7,597 in rural areas).

^b^ The health boundary and range of each food group against 2500 kcal/day in the PHD were converted by the total energy intake in the HSES 2019 (3,085 kcal/day in total, 2,863 kcal/day in urban areas, and 3,529 kcal/day in rural areas).

^c^ Adequacy (%) = dietary intake (g/day) * 100/ health boundary (g/day)

### Dietary intake during two seasons in comparison to the PHD

The differences in dietary intake during the cold and warm seasons compared to the PHD are shown in Table 4. The adequacy of red meat during the cold season (324 g/day, 1,905% adequacy) was higher than that during the warm season (313 g/day, 1,812% adequacy). On the other hand, other consumptions of protein sources, such as chicken and other poultry, fish, and legumes during the cold and warm seasons were lower (0.2–15% adequacy) than those in the PHD, except for eggs (86% adequacy during the cold season and 97% adequacy during the warm season).

**Table 4:** Mongolian dietary intake compared with the health boundary of the planetary health diet and the adequacy during the two seasons.

	Cold seasons			Warm seasons		
Food groups	Dietary intake (g/day)^a^	PHD boundary (range) (g/day)^b^	Adequacy (%)^c^	Dietary intake (g/day)^a^	PHD boundary (range) (g/day)^b^	Adequacy (%)^c^
Whole grains	N.A.	284	N.A.	N.A.	289	N.A.
Tubers or starchy vegetables	94	61 (0, 122)	154	92	62 (0, 123)	148
All vegetables	84	367 (244, 734)	23	85	370 (247, 740)	23
All fruits	31	244 (122, 367)	13	36	247 (123, 370)	15
Dairy foods	231	305 (0, 610)	76	250	309 (0, 617)	81
Protein sources						
Red meat	324	17 (0, 34)	1905	313	17 (0, 35)	1812
Chicken and other poultry	4.2	35 (0, 71)	12	5.4.	36 (0, 72)	15
Egg	14	16 (0, 31)	86	16.	16 (0, 31)	97
Fish	0.6	34 (0, 122)	1.8	0.7	35 (0, 123)	2.0
Legumes	0.2	92 (0, 122)	0.2	0.2	93 (0, 123)	0.2
Nuts	N.A.	61 (0, 92)	N.A.	N.A.	62 (0, 93)	N.A.
Added fats						
Unsaturated oils	19	49 (24, 98)	38	18	50 (25, 100)	36
Saturated oils	7.3	14 (0, 14)	51	7.1	15 (0, 15)	48
Added sugars	47	38 (0, 38)	124	53	38 (0, 38)	137

N.A.: not available, PHD: Planetary Health Diet

^a^ The average dietary intake (g/day) was referenced according to the Household socio-economic survey (HSES) in 2019 among 11,232 households.

^b^ The health boundary and range of each food group against 2,500 kcal/day in the PHD were converted by the total energy intake in the HSES in 2019 (3,057 kcal/day during the cold seasons and 3,111 kcal/day during the warm seasons).

^c^ Adequacy (%) = dietary intake (g/day) * 100/ health boundary (g/day)

### National dietary recommendations in comparison to the PHD

The RDI in Mongolia, compared with the PHD, is presented in Table 5. The three most abundant protein sources were red meat (120 g/day, 1,091% adequacy), eggs (20 g/day, 200% adequacy), and chicken and other poultry (40 g/day, 182% adequacy). The adequacy of other protein sources ranged from 70% to 143%. RDIs in all protein sources were within the range of each PHD, except for red meat (120 g/day in RDI in Mongolia and 0–21 g/day in the PHD). All vegetables and fruits in the RDI in Mongolia were 260 g/day (113% adequacy) and 200 g/day (131% adequacy), respectively, and were within the range of each PHD (153–459 g/day for all vegetables and 77–230 g/day for all fruits).

**Table 5:** Recommended dietary intake in Mongolia compared with the health boundary of the planetary health diet.

Food groups	RDI in Mongolia (g/day)^a^	PHD boundary (range) (g/day)^b^	Adequacy (%)^c^
Whole grains	N.A.	178	N.A.
Tubers or starchy vegetables	120	38 (0, 77)	315
All vegetables	260	230 (153, 459)	113
All fruits	200	153 (77, 230)	131
Dairy foods	340	191 (0, 383)	178
Protein sources			
Red meat	120	11 (0, 21)	1091
Chicken and other poultry	40	22 (0, 44)	182
Egg	20	10 (0, 19)	200
Fish	30	21 (0, 77)	143
Legumes	40	57 (0, 77)	70
Nuts	N.A.	38 (0, 57)	N.A.
Added fats			
Unsaturated oils	23	31 (15, 61)	74
Saturated oils	10	9.0 (0, 9.0)	111
Added sugars	33	24 (0, 24)	137

N.A.: not available, RDI: recommended dietary intake, PHD: planetary health diet

^a^Dietary intake (g/day) was referenced according to the Mongolian standard norms of food.

^b^The health boundary and range of each food group against 2,500 (kcal/day) in the PHD were converted by the total energy intake (2,400 kcal/day) in the RDI in Mongolia.

^c^Adequacy (%) = dietary guidelines (g/day) * 100/ health boundary (g/day)

## Discussion

This study clarified that Mongolian people have an extremely high intake of red meat and a low intake of vegetables and fruits based on the PHD recommendations. These results were more evident in rural areas and during the cold season than in urban areas and during the warm season, respectively. This is the first study to indicate the extent of the discrepancy between the current Mongolian dietary intake and the PHD for planetary and human health.

The present results of high consumption of red meat and low consumption of vegetables and fruits were similar to the global trend [20], except for countries such as India where a vegetarian diet is practiced [10]. Nevertheless, the discrepancy observed in this study was much larger than the global trend [20], Brazil [9], and Denmark [11]. Furthermore, the population intake of red meat, vegetables, and fruits did not meet the RDI in Mongolia in the present study. Among multifactorial interactions, such as limited food availability and accessibility, a lack of nutrition and health knowledge is likely to be a key factor in these results. A review found success in increasing vegetables and fruits intake in many countries by improving nutrition education [21]. A basic and robust system to disseminate nutrition education is required to reach the target of RDI in Mongolia. A study reported that even medical professionals lacked accurate knowledge of the recommended daily salt intake (5 g/day) [22]. Therefore, accurate nutrition education considering sustainable healthy diets to health professionals and communities is required to nudge them to choose healthier and more sustainable food.

This study showed that a high intake of red meat and dairy products and a low intake of vegetables and fruits were more evident in rural areas than urban areas. A similar result was reported in the previous study that a “Nomadic” dietary pattern indicated a high consumption of dairy products, milk, red meat, and refined grains, and low juice and sugar-sweetened beverages, processed meat, and fruit [13]. In addition, the Nomadic pattern was associated with increased iron and zinc intake and decreased fiber intake [13]. Nomadic dietary patterns may result in obesity and a high risk of cardiovascular diseases [6, 7]. According to the geographic characteristics in this study, people living with four or more family members in Ger and owning livestock are likely to have traditional dietary habits. As rural populations practice, traditional Mongolian diets are characterized by a high intake of dairy products (i.e., milk and natural yogurt), fats and oil, sugar, confectionery, and horsemeat [15]. In addition, nomadic herders usually feed themselves, especially on meat and dairy products [13]. These traditional dietary cultures imply that the limited accessibility, availability, and affordability of food retail and variety of food, such as fresh vegetables and fruits, are present in rural areas compared with urban areas.

The environmental impact may differ between urban and rural areas. The GHG emissions from livestock, mainly consumed in urban areas, come from several sources, such as emissions related to feed (e.g., fertilizer and land use), processing, transport, on-farm energy use, and enteric fermentation [23]. The environmental impact generated by livestock and wild game consumed by self-sufficiency in rural areas may be lower than that generated by livestock consumed by people in urban areas, even if there is high consumption of red meat in rural areas.

A higher intake of red meat during the cold season than during the warm season would have a specific background in a severe climate. During the coldest season, the traditional dietary pattern of high red meat (i.e., horsemeat) and fats is usually consumed [24], especially in rural areas, to preserve sufficient energy reserves at severe temperatures [14]. Therefore, the difference in dietary intake between the two seasons should be considered a strategy for sustainable healthy diets.

In this study, animal-based protein sources in the RDI in Mongolia were higher than those in the PHD, particularly red meat. Studies in America and Italy have reported differences between national dietary guidelines and the PHD due to their dietary habits and traditional cultures [25, 26]. A review suggested that national food-based dietary guidelines could be sustainable and healthy to some extent, even if dietary goals are not completely aligned with global health and environmental targets [27]. A new RDI in Mongolia may be required to originally achieve planetary and human health with consideration of feasibility, such as food culture, geographic characteristics, and food variety.

This study did not indicate the intake of whole grains due to the limited information from the HSES. A study reported that the usual mean intake (g/2,500kcal/day) of whole grains (ranging from 2.2 to 20) was lower than that of refined grains (ranging from 361 to 461) in eight provinces in Mongolia [13]. The consumption of refined grains, such as bread, pasta, and rice, may be associated with westernization [15].

The present results are generalizable because this study used an open-source national survey [15]. However, this study has some limitations that warrant mention. First, this study did not include food groups because the lack of information. The intake of whole grains has not been investigated in the HSES and the RDI in Mongolia. Some protein sources in two areas (i.e., chicken and other poultry, eggs, fish, legumes, and nuts) and during the two seasons (i.e., nuts) were investigated but not published in detail. Furthermore, the results of added sugar did not reflect only sugar intake, as this study included confectionery products. The fifth National Nutrition Survey reported the nutritional status of the Mongolian population but did not publish data on food-based dietary consumption [28]. More data should be considered in future studies to clarify the overall status of food intake. Second, the different processes of the 30-day dietary record in urban areas and the 7-day dietary record in rural areas made precise comparison between the two areas difficult. The survey in rural areas was conducted using simple methods compared to urban areas because of the limited resources for the survey, such as manpower. Third, this study did not necessarily adapt Mongolian food culture to the PHD, such as the characteristics of dietary cultures and food availability. The EAT-Lancet Commission recommends the local interpretation and adaptation of the universally applicable PHD [20]. According to the RDI in Mongolia [17], the recommended intake of red meat in the PHD may not be feasible for Mongolian diets. Fourth, the PHD targeted adults aged 18 years and older. Given that the data were available, the interpretation classified by sex and age group (i.e., children and older adults) would differ from the present results. Fourth, the assessment of sustainable healthy diets in Mongolia was indecisive only from this study, using one measurement. The measurement of GHG emissions, water and land use, and nitrogen and phosphorus fertilizer application may help us deeply understand this comprehensive assessment.

## Conclusions

This study indicated an extremely high intake of red meat and a low intake of vegetables and fruits compared to the recommended intake of the PHD among Mongolian people. This discrepancy was larger in rural areas and during the cold season than in urban areas and during the warm season, respectively. To prevent health inequality due to the geographic and seasonal situation of planetary and human health, further policies for multi-sectoral interventions, such as fields of infrastructure and education systems, are required to improve the accessibility, availability, and affordability of healthy food, as well as nutrition education.
